# Multidimensional poverty and health: evidence from a nationwide survey in Japan

**DOI:** 10.1186/s12939-014-0128-9

**Published:** 2014-12-19

**Authors:** Takashi Oshio, Mari Kan

**Affiliations:** Institute of Economic Research, Hitotsubashi University, 2-1 Naka, Kunitachi, Tokyo 186-8603 Japan; School of Economics, University of Hyogo, 8-2-1 Gakuen-Nishi-Machi, Nishiku, Kobe, Hyogo 651-2197 Japan

**Keywords:** Multidimensional poverty, Self-rated health, Psychological distress, Current smoking

## Abstract

**Introduction:**

It is well known that lower income is associated with poorer health, but poverty has several dimensions other than income. In the current study, we investigated the associations between multidimensional poverty and health variables.

**Methods:**

Using micro data obtained from a nationwide population survey in Japan (*N* = 24,905), we focused on four dimensions of poverty (income, education, social protection, and housing conditions) and three health variables (self-rated health (SRH), psychological distress, and current smoking). We examined how health variables were associated with multidimensional poverty measures, based on descriptive and multivariable logistic regression analyses.

**Results:**

Unions as composite measures of multiple poverty dimensions were more useful for identifying individuals in poor SRH or psychological distress than a single dimension such as income. In comparison, intersections of poverty dimensions reduced the coverage of individuals considered to be in poverty and tend to be difficult to justify without any explicit policy objective. Meanwhile, education as a unidimensional poverty indicator could be useful for predicting current smoking.

**Conclusions:**

Results obtained from the current study confirmed the practical relevance of multidimensional poverty for health.

## Introduction

It has been established that health is closely associated with socioeconomic status. Among others, many researchers have found that lower income is associated with poorer health [1–3]. However, it is now widely recognized that poverty is multidimensional rather than unidimensional [4–6]. An individual’s well-being, whether objective or subjective, is likely associated with not only low income but also other poverty dimensions. This is probably also true of health; it is reasonable to expect that health is affected by multiple dimensions of poverty, rather than attributed entirely to a single poverty dimension. Indeed, more attention has been placed on the associations of health with various socioeconomic factors [7,8].

The multidimensional poverty approach originated from Sen’s “capabilities” theory [9]. Sen defines “functionings” as various states of human beings and activities that an individual can undertake. “Capabilities” is defined as an individual’s freedoms or opportunities to choose between different combinations of functionings that he/she has reason to value. In this context, poverty is defined as a lack of freedom due to the deprivation of capabilities and, correspondingly, multidimensional in nature. In recent years, the multidimensional poverty approach has been widely applied to cross-country and country-specific studies of poverty [10–14].

The capabilities theory and multidimensional poverty approach allow us to focus on a particular aspect of life as well as overall well-being, and examine its associations with multiple dimensions of poverty that deprive an individual of capabilities related to that aspect of life. Specifically, we can construct multidimensional poverty measures that could prevent an individual from achieving the capability of enjoying good health and investigate how health is associated with multidimensional poverty.

However, the association between multidimensional poverty and health has been largely understudied. Although many preceding studies have addressed how health is associated with particular poverty dimensions other than income, such as education [14], social protection [15], and housing conditions [16], the multidimensional poverty approach has not been fully applied to health. As one of the first attempts at addressing this issue, Callander, Shofield, and Shrestha [17] examined the association between multidimensional poverty and chronic health conditions using cross-sectional data from Australia. The authors showed that individuals with a chronic health condition were significantly more likely to be in multidimensional poverty than those without. They also observed that a substantial portion of those in multidimensional poverty suffered from a chronic health condition. In addition to Callander et al. [17], recent studies have paid increasing attention to the association between health and multidimensional aspects of social disadvantage and inequalities [18,19], although they did not specifically discuss multidimensional poverty.

In the current study, we investigated how health was related to multidimensional poverty using micro data from a nationwide population survey in Japan (*N* = 24,905). We treated health as an outcome and considered three variables: self-rated health (SRH), psychological distress, and current smoking. The former two represent general and mental health conditions, respectively, while current smoking is a typical health-risk behavior that may mediate the impact of poverty on general health outcomes [20–22]. We focused on four poverty dimensions: income, education, social protection, and housing conditions. Preceding studies have shown that each of the latter three aspects of poverty is independently associated with health even after controlling for income [14–16]. We focused on the evidence obtained from a nationwide population survey in Japan. The relative poverty ratio—i.e., the headcount rate of individuals with income below 50% of the mean income in society—was 16.1% in Japan in 2012, higher than in many other countries in the Organisation for Economic Co-operation and Development (OECD). This implies that the health conditions of Japanese people are under relatively strong pressures from income poverty risks, and it is therefore of great interest to examine to what extent multidimensional poverty implies to health care policies.

## Materials and methods

### Study sample

We used micro data obtained from a nationwide, population-based survey, “Comprehensive Survey of the Living Conditions of People on Health and Welfare” (CSLCPHW), which was conducted and released by the Ministry of Health, Labour and Welfare (MHLW) of the Japanese Government in 2010, with permission from the MHLW. This CSLCPHW survey was authorized by the Ministry of Internal Affairs and Communications (a ministry in charge of all government surveys in Japan) from statistical, legal, ethical, and other viewpoints in accordance with the Statistics Law in Japan. Hence, ethics approval was not required for the current study.

**Figure 1:**
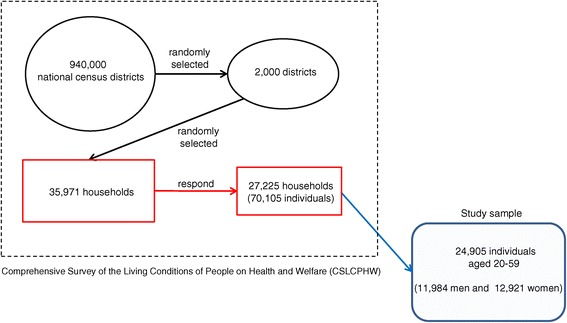
**Construction of the study sample.**

Figure 1 presents a flow diagram to illustrate how the study sample was constructed. Samples were collected nationwide through a two-stage random sampling procedure. First, 2,000 districts were randomly selected from about 940,000 national census districts. Second, 35,971 households were randomly selected from each selected district, according to its population size. All household members of each selected household were asked to complete the questionnaires. A total of 27,225 households and their members (70,105 individuals) responded. The response rate was 75.7% at the household level. Limiting the study sample to individuals aged between 20 and 59 years (due to the reason explained later) and excluding respondents who were missing key variables, we finally used the data of 24,905 individuals (11,984 men and 12,921 women), who made up 76.3% of the total respondents aged between 20 and 59 years in the original sample. Table 1 summarizes the demographic structure of the sample.

**Table 1:** **Demographic characteristics of the sample**

	**Men**	**Women**	**Total**
Proportion (%)			
Age 20s	26.4	26.5	26.5
30s	23.6	22.8	23.2
40s	24.6	25.1	24.9
50s	25.3	25.6	25.5
Never-married	31.7	24.1	27.7
Married	65.0	68.8	67.0
Divorced	0.5	1.6	1.1
Widowed	2.9	5.5	4.2
*N*	11984	12921	24905

### Measures

We constructed multidimensional poverty measures based on a dual-cutoff approach [23–25], as discussed in more detail in the subsection on data analysis. First, on each dimension we defined deprivation as a shortfall from a certain cutoff point. Second, at the aggregated level we defined poverty as a shortfall of the sum of deprivations from a certain cutoff point. As discussed in Atkinson et al. [26], and applied by many preceding studies in multidimensional poverty, we assigned an equal weight to each dimension. We then examined how the results were sensitive to a choice of dimension sets in order to compare the relative importance of each dimension for health.

Specifically, we considered four poverty dimensions: household income, education, social protection, and housing conditions. For household income, we first divided the reported household income by the square root of the number of family members, in order to adjust for household size. This adjustment was based on recent publications by the OECD [27,28]. If an individual’s household-size-adjusted income was below the poverty line (JPY 1.25 million (equivalent to about USD 13,360), which was estimated by the MHLW [29], then he/she was considered to have low income.

For education, an individual was considered to have low educational attainment if his/her highest level of educational attainment was graduation from junior high school. This meant that we set up a cutoff for low educational attainment as nine schooling years. The share of respondents who did not have educational attainment above junior high school was higher for older age groups (4.2% for people in their 20s vs. 8.3% for those in their 50s). We did not adjust this generational difference, considering that raising the cutoff to the graduation from high school would raise the proportion of low educational attainment to above 50%.

Regarding social protection, we used no coverage of public pension insurance as an indicator of its insufficient level. All citizens are required to be covered by a public health insurance program in Japan, but the CSLCPHW survey did not ask respondents about their public health insurance coverage. However, the survey did enquire about public pension insurance coverage. We concentrated on respondents aged between 20 and 59 years old, who are obliged by law to pay public pension premiums, and their responses regarding insurance coverage. Individuals in non-regular employment are less likely to be insured by public pension programs. Indeed, according to the current survey, 5.5% of non-regular employees were uninsured, compared to 0.6% of regular employees.

Finally, we considered housing conditions, which is defined as the amount of living floor space as the fourth dimension of poverty in terms of living standards. The Ministry of Land, Infrastructure, Transport and Tourism (MLIT) defined the minimum living floor space in its Basic Plan for Housing in 2011 as 25 m^2^ for single-person households and (10 + 10 × the number of household numbers) m^2^ for other households, regardless of location (with some adjustment for children) [30]. An individual is considered to live in poor housing conditions if the living floor space per capita of his/her household is below the MLIT-defined minimum level.

We considered self-rated health (SRH), psychological distress, and current smoking as indicators of health. Respondents were asked about their current health conditions on a five-point scale (*good*, *somewhat good*, *average*, *somewhat poor*, and *poor*). We then constructed a binary variable of poor SRH to which we allocated a “1” if the respondent answered *poor* or *somewhat poor*. To measure psychological distress, we employed Kessler 6 (K6) scores [31,32]. We first obtained the respondents’ assessments of psychological distress using a six-item psychological distress questionnaire—“During the past 30 days, approximately how often did you feel a) nervous, b) hopeless, c) restless or fidgety, d) so depressed that nothing could cheer you up, e) that everything required effort, and f) worthless?”— rated on a 5-point scale (0 = *not at all* to 4 = *all of the time*). Then, we calculated the sum of the reported scores (range: 0–24) and defined it as the K6 score. Cronbach’s alpha coefficient was 0.900 for the entire sample. Higher K6 scores reflect higher levels of psychological distress. We then constructed a binary variable of psychological distress to which we allocated a “1” to K6 scores ≥ 5, which indicates a mood/anxiety disorder in a Japanese sample, as established by Sakurai et al. [33]. In addition to these two general health variables, we constructed a binary variable of current smoking, which has been found to be a key mediator between socioeconomic status and health. The prevalence of poor SHR, psychological distress, and current smoking was 11.2%, 31.9%, and 25.4% respectively for the entire sample.

### Data analysis

We first examined whether and to what extent multidimensional poverty is more useful for identifying individuals in poor health compared to unidimensional poverty such as low income. There are two ways of constructing multidimensional poverty measures: union and intersection. Using the terminology of the set theory, a union of sets A (e.g., individuals in low income) and B (e.g., individuals with low education) indicates a set of elements that are in A or in B or in both A and B (individuals with low income or/and low education). An intersection of sets A and B indicates a set of elements that belongs to both A and B (individuals with low income and low education). We expanded these definitions to the cases of more than two sets as discussed below, because we considered a maximum of four poverty dimensions.

There may be a trade-off between the coverage of poor individuals and the association between poverty and health. Although a union of poverty dimensions is likely to widen the coverage of individuals in poverty, it may also reduce the odds of identifying individuals in poor health. In contrast, an intersection of poverty dimensions might raise the odds of identifying individuals in poor health, but would likely be accompanied with a narrower coverage of those in poverty.

Keeping this potential trade-off in mind, we constructed several types of multidimensional poverty based on four poverty dimensions (1 = household income, 2 = education, 3 = social protection, and 4 = housing conditions). To apply a dual-cutoff approach [22–24], we let *D* () indicate the number of deprivations in the poverty dimensions quoted in the parenthesis. Then, we considered an individual to be multidimensionally poor if *D* () was above a certain cutoff point at the aggregated level. For example, *D* (1, 2, 3, 4) ≥ 2 indicates individuals who were deprived in at least two of four dimensions and *D* (2, 3) = 2 indicates individuals who were deprived in both dimensions 2 and 3. This categorization also included unidimensional poverty such as *D* (1) = 1 and *D* (2) = 1.

There were 32 types of multidimensional poverty in total (including four types of unidimensional poverty). Four-dimensional poverty corresponds to *D* (1, 2, 3, 4) ≥ *k*, where *k* = 1, 2, and 3, or *D* (1, 2, 3, 4) = 4. *D* (1, 2, 3, 4) ≥ 1 indicates a union of four dimensions, i.e., a set of individuals who are considered to be poor in at least one of four dimensions. Meanwhile, *D* (1, 2, 3, 4) = 4 indicates their full intersection, i.e., a set of those who are in poverty in all four dimensions. In the current study, we included cases consisting of one to four poverty dimensions.

Based on these multidimensional poverty measures, we first focused on four unidimensional types of poverty, (*D* (1) = 1, …, *D* (4) = 1), and four four-dimensional types of poverty, (*D* (1, 2, 3, 4) ≥ 1, *…*, *D* (1, 2, 3, 4) = 4), and compared the coverage of each type of poverty and proportions of individuals with poor SRH within each poverty type.

Then, we explored how to appropriately define multidimensional poverty, because any methodology based on rigorous theoretical foundations has been not established. It is also difficult to aggregate information on each poverty dimension. If poverty dimensions substantially overlap with one another, then information from many poverty dimensions may be redundant and unidimensional poverty may be more effective. If poverty dimensions were relatively independent from each other, then considering them simultaneously on a multidimensional basis would be more helpful.

To tackle these issues, we estimated logistic regression models to predict poor SRH by a binary variable of each multidimensional poverty along with covariates: gender, age groups (20s (as a reference), 30s, 40s, 50s), and marital status (married (as a reference), never-married, divorced, and widowed). Based on the estimated results, we compared the odds ratios (ORs) of poor SRH, proportions of individuals who were considered to be poor, as well as the goodness of fit across regression models.

We assessed the effectiveness of multidimensional poverty from two aspects, after excluding types of poverty that were not significantly associated with SRH. First, we considered a type of poverty to be an effective predictor of poor SRH if there was no other type of poverty that covered more individuals in poverty and had a higher OR of poor SRH. Second, we compared the likelihood—or more precisely, log-pseudo-likelihood—of the regression model that predicted poor SRH by each type of poverty indicator and the covariates. Because the number of explanatory variables was the same (nine) for all regression models, this assessment was equivalent to that based on the Akaike Information Criteria (AIC). We repeated the same regression analysis for psychological distress in terms of K6 ≥ 5 and current smoking.

## Results

### Descriptive statistics

Table 2 compares the coverage of the four types of unidimensional poverty (top part) and the four types of four-dimensional poverty (bottom) as well as the proportions of individuals with poor SRH, psychological distress (K6 ≥ 5), and current smoking in each poverty type. Although not adjusted for gender, age, and other covariates, this table illustrates the validity and limitations of dimensional poverty.

**Table 2:** **Poverty and poor SRH: a descriptive analysis**

**Dimension of poverty**	**Definition of poverty**	***N***	**Proportion (%) of**			
			**poverty**	**poor SRH**	**K6 ≥ 5**	**current smoking**
1. Household income	*D* (1) = 1	2595	10.4	14.6	34.6	29.0
2. Education	*D* (2) = 1	1303	5.2	15.8	33.3	43.0
3. Social protection	*D* (3) = 1	1077	4.3	17.0	37.2	22.7
4. Housing conditions	*D* (4) = 1	1866	7.5	12.8	34.8	30.2
	*D* (1, 2, 3, 4) ≥ 1	5600	22.5	14.0	35.7	30.2
	*D* (1, 2, 3, 4) ≥ 2	1072	4.3	17.3	38.2	35.1
	*D* (1, 2, 3, 4) ≥ 3	153	0.6	22.9	32.7	31.4
	*D* (1, 2, 3, 4) = 4	16	0.1	31.3	18.8	31.3
All		24905	100.0	11.2	31.9	25.4

*D* denotes the number of deprivation in the dimensions quoted in the parenthesis, where 1 = household income, 2 = education, 3 = social protection, and 4 = housing conditions.

For poor SRH, we first observed that, among the four types of unidimensional poverty, low income, *D* (1) = 1, contained the highest proportion of individuals in poverty (10.4%), with 14.6% perceiving themselves as having poor health, compared to 11.2% of the entire sample reporting poor SRH. Poverty in terms of education, social protection, and housing conditions had lower coverage (4.3–7.5%) but the proportions of individuals with poor SRH in these dimensions (12.8–17.0%) did not differ substantially from that of low income.

Regarding the four types of four-dimensional poverty, we observed that individuals who had at least one of four poverty dimensions, *D* (1, 2, 3, 4) ≥ 1, consisted of 22.5% of the entire sample, with 14.0% of them assessing their health as poor. The proportion of individuals belonging to this category made up more than twice that of low income (*D* (1) = 1), with the number only slightly lower than that for the combined total of the four types of unidimensional poverty (27.4%, not reported in the table). More importantly, the proportion of individuals with poor SRH in this category (14.0%) was comparable to that in the low income dimension (14.6%) and was within the range of that from the other three dimensions (12.8–17.0%).

We also observed that raising the cutoff point above two at the aggregated level reduced the percentage to less than 1%, and a full intersection of poverty dimensions reduced the number of individuals in poverty to only 16 out of 24,905 respondents in the entire sample. Meanwhile, a higher intersection increased the percentage of poor SRH individuals within; a full intersection of the four poverty dimensions increased the percentage to 31.3%.

We found almost similar patterns in results for psychological distress (K6 ≥ 5) and current smoking, while the trade-off between the coverage and intersection of poverty was less clear for these two health variables. A higher intersection than two lowered rather than raised the proportions of K6 ≥ 5 and current smoking.

### Regression analysis

Table 3 presents the estimated results obtained from separately estimated logistic regression models, after controlling for covariates (gender, age, and marital status). The second column indicates the coverage of individuals in poverty, while the third and fourth columns report the odds ratios (OR) of poor SRH in response to each type of poverty and their 95% confidence intervals (CI), respectively.

**Table 3:** **The estimated associations between different types of poverty and poor self-rated health (SRH)**

**Type of poverty**	**Proportion (%) of poverty**	**OR**	**95% CI**	**Effective or not**	**Log likelihood**	**[Rank]**
*D* (1) = 1	10.4	1.38	(1.23, 1.56)		−8666.56	
*D* (2) = 1	5.2	1.49	(1.27, 1.74)		−8668.68	
*D* (3) = 1	4.3	1.89	(1.59, 2.24)	Effective	−8656.30	[3]
*D* (4) = 1	7.5	1.22	(1.06, 1.40)		−8676.76	
*D* (1, 2) ≥ 1	14.4	1.47	(1.33, 1.63)		−8676.76	
*D* (1, 3) ≥ 1	13.7	1.51	(1.36, 1.68)		−8653.19	
*D* (1, 4) ≥ 1	16.4	1.31	(1.18, 1.45)		−8667.23	
*D* (2, 3) ≥ 1	9.0	1.57	(1.39, 1.78)	Effective	−8656.70	[4]
*D* (2, 4) ≥ 1	12.0	1.32	(1.18, 1.48)		−8668.99	
*D* (3, 4) ≥ 1	11.3	1.43	(1.28, 1.61)		−8663.13	
*D* (1, 2, 3) ≥ 1	17.3	1.52	(1.38, 1.68)	Effective	−8646.36	[1]
*D* (1, 2, 4) ≥ 1	19.9	1.38	(1.26, 1.52)		−8658.13	
*D* (1, 3, 4) ≥ 1	19.3	1.40	(1.27, 1.53)		−8657.71	
*D* (2, 3, 4) ≥ 1	15.3	1.41	(1.27, 1.56)		−8660.08	
*D* (1, 2, 3, 4) ≥ 1	22.5	1.42	(1.30, 1.56)	Effective	−8651.76	[2]
*D* (1, 2) = 2	1.2	1.31	(0.95, 1.80)		−8678.95	
*D* (1, 3) = 2	1.1	2.14	(1.58, 2.89)		−8669.81	
*D* (1, 4) = 2	1.5	1.64	(1.25, 2.16)		−8674.66	
*D* (2, 3) = 2	0.6	3.42	(2.38, 4.90)	Effective	−8661.59	[5]
*D* (2, 4) = 2	0.7	1.75	(1.19, 2.56)		−8676.59	
*D* (3, 4) = 2	0.6	2.17	(1.43, 3.28)		−8674.50	
*D* (1, 2, 3) ≥ 2	2.5	1.77	(1.43, 2.19)		−8667.77	
*D* (1, 2, 4) ≥ 2	3.1	1.49	(1.22, 1.82)		−8673.22	
*D* (1, 3, 4) ≥ 2	2.7	1.92	(1.57, 2.34)	Effective	−8662.90	[7]
*D* (2, 3, 4) ≥ 2	1.6	2.21	(1.73, 2.82)	Effective	−8662.75	[6]
*D* (1, 2, 3, 4) ≥ 2	4.3	1.73	(1.46, 2.04)		−8661.46	
*D* (1, 2, 3) = 3	0.2	3.37	(1.86, 6.11)		−8673.24	
*D* (1, 2, 4) = 3	0.2	1.94	(0.99, 3.82)		−8678.57	
*D* (1, 3, 4) = 3	0.2	1.97	(1.04, 3.74)		−8678.33	
*D* (2, 3, 4) = 3	0.1	3.49	(1.61, 7.56)	Effective	−8675.91	[9]
*D* (1, 2, 3, 4) ≥ 3	0.6	2.37	(1.62, 3.48)	Effective	−8671.92	[8]
*D* (1, 2, 3, 4) = 4	0.1	3.11	(1.01, 9.54)		−8678.40	

The OR indicates the estimated odds ratio of poor SRH, obtained from the logistic regression models to predict poor SRH by poverty and covariates (sex, age, and marital status). *D* denotes the number of deprivation in the dimensions in the subsequent parenthesis, where 1 = household income, 2 = education, 3 = social protection, and 4 = housing conditions. Rank indicates the ranking order of pseudo log likelihood.

The association between poverty with poor SRH in terms of OR was significant at the 5% level for most definitions of poverty, with two exceptions: *D* (1, 2) = 2 and *D* (1, 2, 4) = 3. Increasing the cutoff point at the aggregated level, i.e., defining multidimensional poverty as a higher intersection of poverty dimensions, tended to increase the OR, in line with the results in Table 1. At the same time, increasing the cutoff points to above one substantially reduced the proportion of individuals considered to be in poverty.

Applying our definition of effectiveness to all types of poverty, we identified nine effective types of poverty, indicated in the fifth column of Table 3; for example, *D* (1) = 1, which covered 10.4% of the entire sample with an OR of 1.38 was not effective, because *D* (1, 2) ≥ 1 covered a larger percentage (14.4%) and had a higher OR (1.47). From this result, we first observed that three out of four types of unidimensional poverty—somewhat surprisingly including low income (*D* (1) = 1)—were not effective in predicting SRH, underscoring the validity of multidimensional poverty. Second, we found that among the nine effective types of poverty, there was a clear tradeoff between the coverage of individuals in poverty and OR of poor SRH.

Table 3 ranks the likelihood values of the nine indicators of poverty in descending order. The likelihood of predicting poor SRH was highest for *D* (1, 2, 3) ≥ 1. We also noticed that the likelihood tended to be higher for unions than intersections of poverty dimensions.

**Figure 2:**
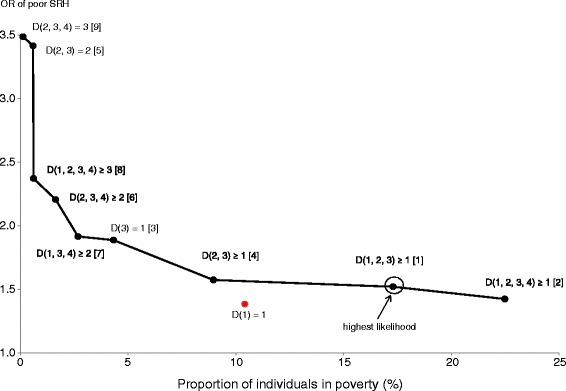
**The effective poverty curve for poor self-rated health (SRH).** Dots on the line indicate a combination of the proportion of individuals in poverty and the odds ratio (OR) of poor SRH for each effective type of poverty. Figures in brackets indicate the ranking order of likelihood of predicting poor SRH.

To graphically illustrate a preferred choice of poverty dimension, Figure 2 depicts the “effective poverty curve,” which plots a combination of the coverage of individuals in poverty and the OR of poor SRH for nine types of poverty that effectively predicted poor SRH. The effective poverty curve has a downward slope, reflecting the trade-off between the coverage of individuals in poverty and the OR of poor SRH. For reference, we added a dot corresponding to income as unidimensional poverty, *D* (1) = 1. This lies below the effective poverty curve, indicating its ineffectiveness. The dot corresponding to *D* (1, 2, 3) ≥ 1, which had the highest predictive power, was circled.

It is relatively easy to compare poverty types located on the relatively horizontal portion of the effective poverty curve. For example, among the three types of poverty (*D* (2, 3) ≥ 1, *D* (1, 2, 3) ≥ 1, and *D* (1, 2, 3, 4) ≥ 1) in Figure 1, all of which are located on such an area, we consider *D* (1, 2, 3) ≥ 1, which has the highest likelihood, as a reference point. Moving rightward from *D* (1, 2, 3) ≥ 1 to *D* (1, 2, 3, 4) ≥ 1 is acceptable in practice, because it leads to increased coverage with limited decline in OR and likelihood. In comparison, moving leftward to *D* (2, 3) ≥ 1 can be hardly justified, because it leads to a substantial decline in coverage with a limited increase in OR. Hence, among the three abovementioned types of poverty, *D* (1, 2, 3) ≥ 1 and *D* (1, 2, 3, 4) ≥ 1 are preferred to *D* (2, 3) ≥ 1.

In contrast, it is difficult to choose from between poverty types located in the upper left and those located in the lower right of the effective poverty curve. The former, which reflected higher intersections of dimensions, had higher ORs than the latter. This, although technically preferred, reduces coverage and likelihood. This is because increasing the cutoff points decreases the possibility of identifying individuals who are at similar risks of poor SRH but were not considered poor enough. However, if the policy priority is on identifying individuals most likely to experience poor SRH, then a combination of low coverage and high ORs may be accepted.

**Figure 3:**
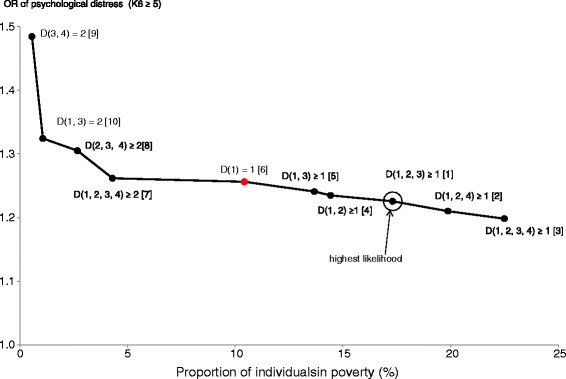
**The effective poverty curve for psychological distress (K6 ≥ 5).** Dots on the line indicate a combination of the proportion of individuals in poverty and the odds ratio (OR) of K6 ≥ 5 for each effective type of poverty. Figures in brackets indicate the ranking order of likelihood of predicting K6 ≥ 5.

**Figure 4:**
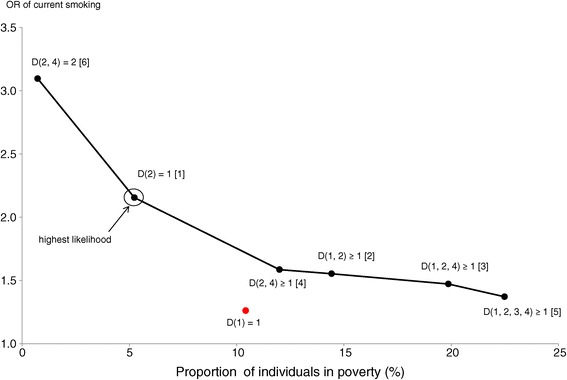
**The effective poverty curve for current smoking.** Dots on the line indicate a combination of the proportion of individuals in poverty and the odds ratio (OR) of current smoking for each effective type of poverty. Figures in brackets indicate the ranking order of likelihood of predicting current smoking.

Table 4 presents the results for psychological distress and current smoking, both of which replaced poor SRH as the key explanatory variable. The table presents the results of only the effective types of poverty. Similar to Figure 2, Figures 3 and 4 graphically illustrate the effective poverty curves for psychological distress and current smoking, respectively.

**Table 4:** **The estimated associations between multidimensional poverty and health**

**Type of poverty**	**Proportion (%) of poverty**	**OR**	**95% CI**	**Log likelihood**	**[Rank]**
Health variable = psychological distress (K6 ≥ 5)					
*D* (1, 2, 3, 4) ≥ 1	22.5	1.20	(1.12, 1.28)	−15495.46	[3]
*D* (1, 2, 4) ≥ 1	19.9	1.21	(1.13, 1.29)	−15495.03	[2]
*D* (1, 2, 3) ≥ 1	17.3	1.23	(1.14, 1.31)	−15494.82	[1]
*D* (1, 2) ≥ 1	14.4	1.23	(1.15, 1.63)	−15495.78	[4]
*D* (1, 3) ≥ 1	13.7	1.24	(1.15, 1.33)	−15495.87	[5]
*D* (1) = 1	10.4	1.26	(1.15, 1.34)	−15497.32	[6]
*D* (1, 2, 3, 4) ≥ 2	4.3	1.26	(1.11, 1.43)	−15504.55	[7]
*D* (2, 3, 4) ≥ 2	1.6	1.31	(1.11, 1.53)	−15504.54	[8]
*D* (1, 3) = 2	1.1	1.32	(1.04, 1.69)	−15508.37	[10]
*D* (3, 4) = 2	0.6	1.48	(1.06, 2.07)	−15508.20	[9]
Health variable = current smoking					
*D* (1, 2, 3, 4) ≥ 1	22.5	1.37	(1.28, 1.47)	−13021.19	[5]
*D* (1, 2, 4) ≥ 1	19.9	1.47	(1.37, 1.58)	−13005.39	[3]
*D* (1, 2) ≥ 1	14.4	1.55	(1.43, 1.69)	−13004.37	[2]
*D* (2, 4) ≥ 1	12.0	1.59	(1.45, 1.73)	−13006.00	[4]
*D* (2) = 1	5.2	2.15	(1.90, 2.44)	−12986.05	[1]
*D* (2, 4) = 2	0.7	3.10	(2.20, 4.36)	−13035.14	[6]
*cf. D* (1) = 1	10.4	1.24	(1.15, 1.39)	−13048.23	–

The OR indicates the estimated odds ratio of poor SRH, obtained from the logistic regression models to predict poor SRH by poverty and covariates (sex, age, and marital status). *D* denotes the number of deprivation in the dimensions in the subsequent parenthesis, where 1 = household income, 2 = education, 3 = social protection, and 4 = housing conditions. Rank indicates the ranking order of pseudo log likelihood.

Figure 3 can help assess the effectiveness of types of poverty for psychological distress. Moving rightward from *D* (1, 2, 3) ≥ 1 to *D* (1, 2, 4) ≥ 1, or even to *D* (1, 2, 3, 4) ≥ 1 can be accepted, because doing so increases coverage with limited loss in OR and likelihood. In contrast, intersections of poverty dimensions increases OR but reduces coverage and likelihood, a pattern similar to poor SRH. However, the estimated ORs of the intersections lay within a more limited range (1.26–1.48) compared to that of poor SRH (1.42–3.49). This means that increasing the cutoff points to 2 (i.e., moving toward the upper left) was less justifiable without any specific policy objective for psychological distress than for poor SRH.

Lastly, we noticed that the effective poverty curve for current smoking (Figure 4) showed differences from those of poor SRH and psychological distress. Most notably, we found that low education, rather than low income, was the key poverty dimension for current smoking. The unidimensional poverty of low education, *D* (2) = 1, was effective and had the highest likelihood. Moreover, all of the six effective types of poverty included education as a poverty dimension, underscoring the effectiveness of focusing on education. In addition, it is difficult to justify the union of education and housing conditions, *D* (2, 4) = 2, because it reduced coverage to below 1% and substantially reduced the likelihood. Moving from *D* (2) = 1 to *D* (1, 2) ≥ 1 or *D* (1, 2, 4) ≥ 1 may be acceptable considering their relatively high likelihood; however, we cannot avoid a substantial decline in the OR. Compared to these two types, *D* (2, 4) ≥ 1 and *D* (1, 2, 3, 4) ≥ 1 were less attractive, because of their lower coverage and OR, respectively, as well as their lower likelihood.

## Discussion

The results of the current study underscored the validity of multidimensional poverty in identifying individuals experiencing poor health. When using SRH as a health outcome, multidimensional poverty defined by a union of three or four poverty dimensions (income, education, social protection, and housing) was preferred to low income and other unidimensional poverty types.

However, it is difficult to determine which type of poverty was the most appropriate for predicting SRH; this depends on the policy objective, or more broadly, value judgment. For example, if we wanted to identify individuals who face the highest risks of poor SRH, then the most appropriate poverty indicator would be *D* (2, 3, 4) = 3, which had the highest OR (3.49) among the nine types of poverty; however, this indicator covered only 0.1% of the total population. In contrast, if we wanted to identify individuals facing higher-than-average risks of poor SRH as widely as possible, then *D* (1, 2, 3, 4) ≥ 1 would be more appropriate, covering 22.5% of the population, albeit with a relatively low OR (1.42).

In the case of psychological distress measured by K6 scores, we observed both similarities and differences compared to those for poor SRH. First, among the ten types of poverty that could effectively predict psychological distress, *D* (1, 2, 3) ≥ 1 had the highest likelihood, similar to that for poor SRH. Second, the likelihood of the model tended to be higher for unions rather than intersections of poverty dimensions, a pattern similar to that observed for poor SRH. Third, compared to poor SRH, psychological distress had a closer association with low income. Eight of the ten types of poverty included income as a relevant poverty dimension, and unidimensional poverty for income, *D* (1) = 1, was assessed to be effective. Fourth, more types of poverty were located on the relatively horizontal portion of the effective poverty curve: from *D* (1, 2, 3, 4) ≥ 2 to *D* (1, 2, 3, 4) ≥ 1.

For both SRH and psychological distress, we noticed that the intersections of income and other dimensions led to a substantial reduction in coverage of individuals in poverty, consistent with results from the United States [22]. This fact reflected a limited overlap among the dimensions and implies that the cutoff point at the aggregate level in a dual-cutoff approach should not be too high. Actually, we found that a cutoff point of one was preferable in many cases for both SRH and psychological distress.

Meanwhile, we can suspect that multidimensional poverty may be less effective in identifying individuals who have specific health-risk behaviors. As an illustrative example, we showed that low educational attainment was the single key correlate to current smoking. This result is consistent with observations in many preceding studies that reported close associations between education and smoking [34–36]. While we should be cautious with any generalization, it is reasonable to predict that the more specific health-risk behaviors—or more broadly, health variables—are, the more closely they are related to poverty in specific dimensions.

It should be noted, however, that the choice of multidimensional poverty measures depends heavily on policy objectives. From among the possible combinations of multidimensional poverty, we can select the most desirable types of poverty, which can be performed through plotting the effective poverty curve. However, choosing the ideal poverty indicator from this subset constitutes a tradeoff between the coverage of individuals in poverty and the probability of finding individuals with poor health. As far as general health conditions are concerned, our results suggest that reducing coverage by increasing the cutoff points at the aggregated level, i.e., targeting higher intersections of poverty dimensions, would be costly; doing so would likely fail to capture individuals who are at the highest risk of poor health.

### Limitations and future research issues

We acknowledge that the current studies have several limitations. First, we could not establish causality between multidimensional poverty and health, because our analysis was based on cross-sectional data. For example, we cannot rule out the possibility that poor health conditions increase the risk of having a low income, which in turn negatively affects other poverty dimensions.

Second, we used only binary variables for all poverty dimensions, which did not capture the depth of poverty. For example, we defined low income as household income below the poverty line. This definition disregarded differences in the degree of poverty across individuals whose income was below the poverty line [22,23]. The same problem applies for health variables. We also constructed binary variables for SRH (originally on a five-point score) and psychological distress (based on continuous K6 scores). Our regression analysis was based on cutoff points widely used in preceding studies, but we should be cautious when generalizing the estimated magnitudes of the associations, which may be affected by choices of the cutoff points.

Third, we should examine the sensitivity of the results to the choice of relative weights assigned to poverty dimensions. Instead of performing any normative value judgment, the current study assigned an equal weight to each poverty dimension and explored how a choice of dimensions affected the effectiveness of multidimensional poverty measures. However, it is of interest to investigate how different weights would affect the results, especially if there are any compelling reasons to consider one dimension to be more important than another.

Fourth, we should expand the current study to examine how sex and other individual attributes confound the association between multidimensional poverty and health. Our regression analysis controlled for sex, age, and marital status by including them as covariates, but their interactions with multidimensional poverty were not investigated. This is especially true for sex, which most likely affects the association with smoking.

Lastly, the mechanism that links multidimensional poverty and health remains to be uncovered. Based on Sen’s capabilities theory [10], we focused on four poverty dimensions that are expected to prevent an individual from achieving favorable health outcomes. Then, we examined how multidimensional poverty, which was constructed from these dimensions, was associated with health. However, the importance of the pathway between multidimensional poverty and health was suggested by a difference in results between general health conditions (SRH and psychological distress) and a specific health-risk behavior (current smoking). These issues should be addressed in future research.

## Conclusions

We confirmed the validity of multidimensional poverty in identifying individuals with poor health using micro data from a nationwide survey in Japan. Unlike most of preceding studies that focused on the association with health and specific aspects of poverty, the current study investigated how health was related to multidimensional poverty. We observed that unions of multiple poverty dimensions were more useful for identifying individuals with poor SRH or psychological distress than a single dimension such as income. In comparison, intersections of poverty dimensions reduced the coverage of individuals considered to be in poverty and are difficult to justify without any explicit policy objective. Meanwhile, when focusing on the unidimensional poverty, education could be justified for current smoking. These findings provided new insight into the discussion on the social determinants of health.

In sum, the results obtained from the current study underscored the practical relevance of multidimensional poverty for public health. Policymakers should consider multiple dimensions of poverty rather than focusing exclusively on income or other single dimension, especially if they aim at improving people’s general or mental health conditions. Even if policymakers tackle specific health problems, the multidimensional poverty approach is expected to help them identify which aspect of poverty or combination of poverty aspects that they should consider most seriously.

## Abbreviations

CSLCPHW: Comprehensive Survey of the Living Conditions of People on Health and Welfare
K6: Kessler 6
MHLW: Ministry of Health, Labour and Welfare
MLIT: Ministry of Land, Infrastructure, Transport and Tourism
MPI: Multidimensional Poverty Index
OECD: Organisation for Economic Co-operation and Development
OPHI: Oxford Poverty and Human Development Initiative
SRH: Self-rated health
UNDP: United Nations Development Programme
